# Rapid unpaired CBCT‐based synthetic CT for CBCT‐guided adaptive radiotherapy

**DOI:** 10.1002/acm2.14064

**Published:** 2023-06-22

**Authors:** Jacob F. Wynne, Yang Lei, Shaoyan Pan, Tonghe Wang, Mosa Pasha, Kirk Luca, Justin Roper, Pretesh Patel, Sagar A. Patel, Karen Godette, Ashesh B. Jani, Xiaofeng Yang

**Affiliations:** ^1^ Department of Radiation Oncology and Winship Cancer Institute Emory University Atlanta Georgia USA; ^2^ Department of Medical Physics Memorial Sloan Kettering Cancer Center New York New York USA

**Keywords:** adaptive radiotherapy, machine learning, deep learning, image synthesis, image translation

## Abstract

In this work, we demonstrate a method for rapid synthesis of high‐quality CT images from unpaired, low‐quality CBCT images, permitting CBCT‐based adaptive radiotherapy. We adapt contrastive unpaired translation (CUT) to be used with medical images and evaluate the results on an institutional pelvic CT dataset. We compare the method against cycleGAN using mean absolute error, structural similarity index, root mean squared error, and Frèchet Inception Distance and show that CUT significantly outperforms cycleGAN while requiring less time and fewer resources. The investigated method improves the feasibility of online adaptive radiotherapy over the present state‐of‐the‐art.

## 1 INTRODUCTION

Radiation therapy is an essential tool in the treatment of prostate cancer. Modern treatment approaches are increasingly hypofractionated,^1^, ^2^, ^3^, ^4^ delivering greater doses in each fraction relative to historical regimens. Greater doses have been associated with increases in biochemical progression‐free survival^4^, ^5^ as well as reductions in prostate cancer‐specific mortality^4^ and distant metastases.^4^, ^5^ Accurate targeting is a critical challenge which must be overcome to safely deliver ablative doses to pelvic targets while sparing nearby organs at risk (OARs). Image‐guided radiotherapy (IGRT) is a treatment strategy that uses imaging to monitor variation in the anatomical position of tumor targets and OARs to ensure appropriate target coverage while avoiding nearby healthy tissues. Adaptive radiation therapy, wherein IGRT treatment plans are revised to account for setup error and physiologic motion (e.g., variable bladder filling, peristalsis) prior to the delivery of each dose fraction, is an emerging strategy to address this challenge. While specialized equipment has been developed to provide diagnostic‐quality fan‐beam X‐ray computed tomography (FBCT) and magnetic resonance imaging (MRI) in this setting, X‐ray cone‐beam CT (CBCT) is broadly available on modern radiotherapy equipment and is more commonly used for this purpose. However, due to increased scatter resulting from greater detector size, limited beam collimation, and lower beam energies, CBCT suffers from greater noise, more prominent artifacts, and inaccurate Hounsfield unit (HU) values relative to diagnostic CT. Resultant loss of soft tissue contrast compromises the accuracy of patient position verification while HU inaccuracy limits dosimetric utility. Dosimetric fidelity is particularly crucial for patients, like those in this study, being treated with proton therapy where inaccurate calculation of relative stopping power may add to range uncertainty resulting in suboptimal target coverage.

Several solutions have been proposed to improve CBCT quality. Monte Carlo (MC) methods simulate physical photon transport within tissues with mathematical rigor, but computational inefficiency precludes routine clinical use.^6^, ^7^, ^8^ Scatter correction algorithms seek to approximate the physical scatter of photons recorded in CT projection data by assuming scatter signal can be represented by a convolutional function of the signal with a scatter kernel.^9^, ^10^ These methods often rely on underlying MC models and are subject to their associated inefficiencies. Histogram matching strategies apply a linear function to individual pixel values of CBCT to estimate those of a diagnostic‐quality planning CT.^11^, ^12^, ^13^

Machine learning provides several advantages over other methods. Computationally burdensome models of underlying physical phenomena may be circumvented by taking images as inputs and generating images as outputs, exploiting the efficiency of modern graphics processing units and allowing experiments to be conducted on consumer‐grade devices. Most machine learning methods published to date^14^, ^15^, ^16^, ^17^, ^18^, ^19^, ^20^ are based on the cycle‐consistent generative adversarial network (CycleGAN).^21^ CycleGAN allows training on unpaired image data by introducing a cycle‐consistency loss, which constrains solution optimization, improving the stability of training and speed of convergence. In the setting of medical imaging, where manually annotated ground truth data are often not available, such an approach is advantageous. However, because CycleGAN implicitly enforces bijection, it frequently suffers from mode collapse producing only one output regardless of model input.^22^ Because CycleGAN was initially designed for use with natural images, the architecture may also fail to preserve anatomical boundaries. Several solutions to these problems have been investigated. Paired images have been provided as inputs to improve boundary preservation^15^, ^16^ and the full CT HU range ([‐1024, 3071]) has been variably clipped to improve model training.^16^, ^18^ HU clipping makes CBCT‐based dose calculation impossible and limits evaluation of the model on the entire range of human tissues. Without simultaneous CBCT and FBCT, these studies utilizing paired inputs have also used various strategies based on deformable image registration (DIR) of planning CT to approximate ground truth images representing the actual position of patient anatomy and evaluate their results. To our knowledge, only two other groups have demonstrated unpaired CBCT synthesis with HU fidelity and anatomic boundary preservation required for clinical deployment in an adaptive radiotherapy (ART) workflow.^14^, ^23^

We investigate a method for image‐to‐image translation from CBCT to FBCT using contrastive unpaired translation.^24^ Our method takes unpaired CBCT and FBCT data as inputs and generates FBCT‐quality synthetic CT images as outputs. We train our model on an institutional dataset of pelvic CT images. To evaluate the result, we compare metrics of image quality, including the mean absolute error (MAE), structural similarity index measure (SSIM), and root‐mean‐square error (RMSE) as well as Fréchet inception distance (FID) relative to same‐day quality assurance FBCT. The method presented here improves upon prior methods by demonstrating anatomic boundary preservation and HU fidelity superior to cycleGAN while significantly reducing compute time^14^ and is evaluated against same‐day FBCT, a more rigorous performance benchmark that eliminates the error introduced when evaluating against deformably‐registered planning CT images.^23^ These improvements support the utility of this technique in an ART workflow.

## 2 METHODS

### 2.1 Data acquisition and processing

Same‐day CBCT and FBCT images acquired from 79 patients receiving proton therapy for prostate cancer between 2019 and 2020 at the Emory Proton Therapy Center of the Emory University School of Medicine in Atlanta, Georgia, USA were retrospectively collected from an institutional database (IRB00114349). FBCT images in this dataset were acquired for the purpose of routine quality assurance, in accordance with institutional policy. Seventy‐nine patients yielded 102 non‐contrast CBCT‐FBCT image pairs with 6 patients undergoing 3 replans and 11 patients undergoing 2 replans during their treatment course. Each QACT image was registered to the corresponding CBCT and resampled to 1 × 1 × 2 mm to establish uniform voxel size and spacing. Images were randomly shuffled prior to input for unsupervised training. The model was trained on the full‐sized 512 × 512 × 104 CT images. A binary mask was generated to remove non‐anatomical regions (treatment couch) from the images. These were generated by applying Otsu's auto‐threshold.^25^ To preserve HU fidelity while maintaining the computational advantages of the existing methods in the published codebase, the full HU data range [‐1024, 3071] was partitioned into equal three segments ([‐1024, 341], [341, 1706], [1706, 3071]), which were each rescaled to [0, 255] and distributed among three RGB channels with 8‐bit depth.

Experiments were conducted on a computer workstation equipped with a single 12 GB NVIDIA TITAN Xp GPU running CUDA 11.7 and a 3.0 GHz Intel Xeon E5‐2623V3 CPU with 32 GB memory on Ubuntu 20.04.4 LTS. The presented architecture was implemented in PyTorch 1.4.0 using Python 3.7.0. Training requires approximately 2 h per epoch. Models were trained with a learning rate of 0.2 for 3 epochs with a subsequent decay to 0.0 over the remaining 3 epochs. For the CUT model, translation requires approximately 19 s for one CT image volume at 5.6 slices per second.

A public dataset was collected from The Cancer Imaging Archive^26^ and comprises approximately 88 CBCT and 130 FBCT images collected from 58 patients treated at the Beaumont Proton Center of Oakland University's William Beaumont School of Medicine Rochester Hills, Auburn Hills, Michigan, USA. Each patient received a planning CT on a 16‐slice Philips Brilliance Big Bore CT scanner (Philips NA Corp, Andover, Massachusetts, USA) covering the entire anatomic region and utilizing an immobilization system. Each patient had CBCT images acquired for daily image guidance on the ProteusONE Proton therapy machine (Ion Beam Applications S.A., Belgium). The CBCT images were 768 × 768 × 110 voxel with voxel size ranging from (0.6406 × 0.6406) to (0.5176 × 0.5176) mm^2^ and 2.5 mm slice thickness for all cases. The planning CT was resampled to the same dimensions in the X/Y plane as the CBCT and the image content was shifted to place the anatomic isocenter at the center of the planning target volume.^27^

### 2.2 Model

#### 2.2.1 Contrastive unpaired translation (CUT)

**FIGURE 1:**
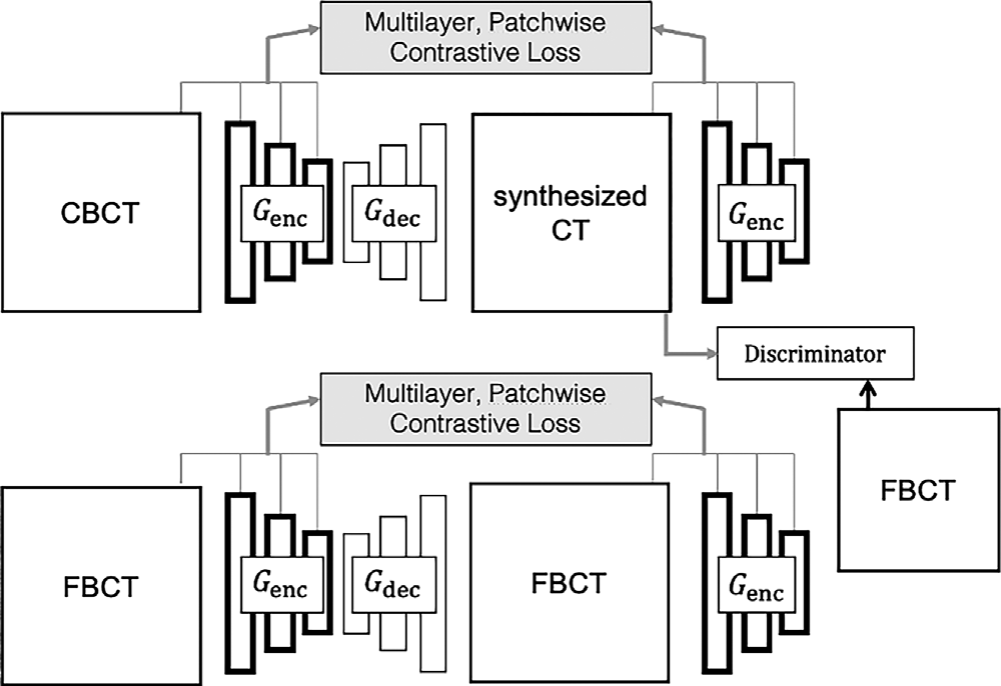
CUT architecture.

A standard GAN^28^ comprises two competing networks, a generator and discriminator, which are trained simultaneously: the generator outputs realistic images approximating those belonging to the target domain while the discriminator works to differentiate these from real images from that domain. CycleGAN^21^ introduces an inverse mapping in the opposite direction and enforces a cycle‐consistency loss to further constrain the mapping, improving network stability during training, and allowing for unpaired image translation. CUT^24^ borrows generator and discriminator architectures from GAN and CycleGAN; however, unlike GAN and CycleGAN, which operate on entire images, CUT introduces a multi‐layer patch‐based approach that maximizes mutual information between image regions by drawing negatives from within the input image rather than from other images in the dataset. CUT therefore requires fewer networks and parameters, improving computational efficiency. Interested readers may refer to Park et al.’s publication^24^ for greater detail regarding the network design, which is summarized in Figure 1.

#### 2.2.2 Loss formulation

A standard adversarial loss^28^ encourages visual similarity of outputs to images in the target domain. To preserve anatomical structure and tissue boundaries, a multi‐layer patch‐wise noise contrastive estimation (PatchNCE) framework is employed, which maximizes mutual information between the input and output. The contrastive approach seeks to associate a “query” patch from the output and its matching “positive” input while dissociating from the remaining non‐matching “negative” inputs in the dataset. The task is thus formulated as an (N+1)‐way classification task. The distances between the query and the other patches are incorporated into a cross‐entropy loss representing the probability of the positive being selected over the negatives. Each layer and location within the feature stack of the encoder $G_{enc}$ represents a patch of the input image, with deeper layers corresponding to larger patches. This feature stack is exploited to further constrain the model and increase the input image signal. *L* layers of interest are selected and their feature maps are passed through a two‐layer multi‐layer perceptron network, yielding a stack of features as in SimCLR.^29^ Patches within the input, rather than across the dataset, are selected as negatives yielding the PatchNCE loss.^24^

The final minimax learning objective (Equation 1) incorporates an equally weighted $\left(\lambda_{X}=\lambda_{Y}=1\right)$ PatchNCE loss on images from the *y* domain to prevent the generator from making unnecessary changes and represents a domain‐specific identity loss:

(1) $$\begin{array}{ccc} & & L_{\mathrm{GAN}}\left(G,\;D,\;X,\;Y\right)+\lambda_{X}L_{\mathrm{PatchNCE}}\left(G,\;H,\;X\right)\\ & & \;+\;\lambda_{Y}L_{\mathrm{PatchNCE}}\left(G,\;H,\;Y\right) \end{array}$$

### 2.3 Evaluation

To evaluate the performance of the CUT model, the output is registered to the quality assurance FBCT at test time and compared using MAE (Equation 2), RMSE (Equation 3), SSIM (Equation 4), and FID. MAE measures the average absolute error of pixels occupying the same position across two images and is therefore reliant upon accurate image registration and reported in HU:

(2) $$MAE=\;\frac{\sum_{i=1}^{n}\left|Y_{i}-X_{i}\right|}{n}$$

RMSE is similarly the quadratic mean of the pixel‐wise errors across images, reported in HU:

(3) $$RMSE=\sqrt{\frac{\sum_{i=1}^{n}{\left(Y_{i}-X_{i}\right)}^{2}}{n}}$$

SSIM is a unitless weighted comparison of luminance, contrast, and structure.^30^ When the weights are set uniformly to 1, as they are here, SSIM reduces to:

(4) $$SSIM\left(x,y\right)=\frac{\left(2\mu_{x}\mu_{y}+c_{1}\right)\left(2\sigma_{xy}+c_{2}\right)}{\left(\mu_{x}^{2}+\mu_{y}^{2}+c_{1}\right)\left(\sigma_{x}^{2}+\sigma_{y}^{2}+c_{2}\right)}$$

where $\mu_{x}$ is the arithmetic mean of *x*, $\mu_{y}$ is the arithmetic mean of *y*, $\sigma_{x}^{2}$ is the variance of *x*, and $\sigma_{y}^{2}$ is the variance of *y*.

FID compares the distribution of generated images with the distribution of real images used to train the generator and is the standard metric by which to assess the quality of generative models. It compares these distributions in the latent space after the generated and real images reach the deepest layer of an Inception v3 model trained on ImageNet,^31^ the details of which are described in the original publication.^32^

## 3 RESULTS

**FIGURE 2:**
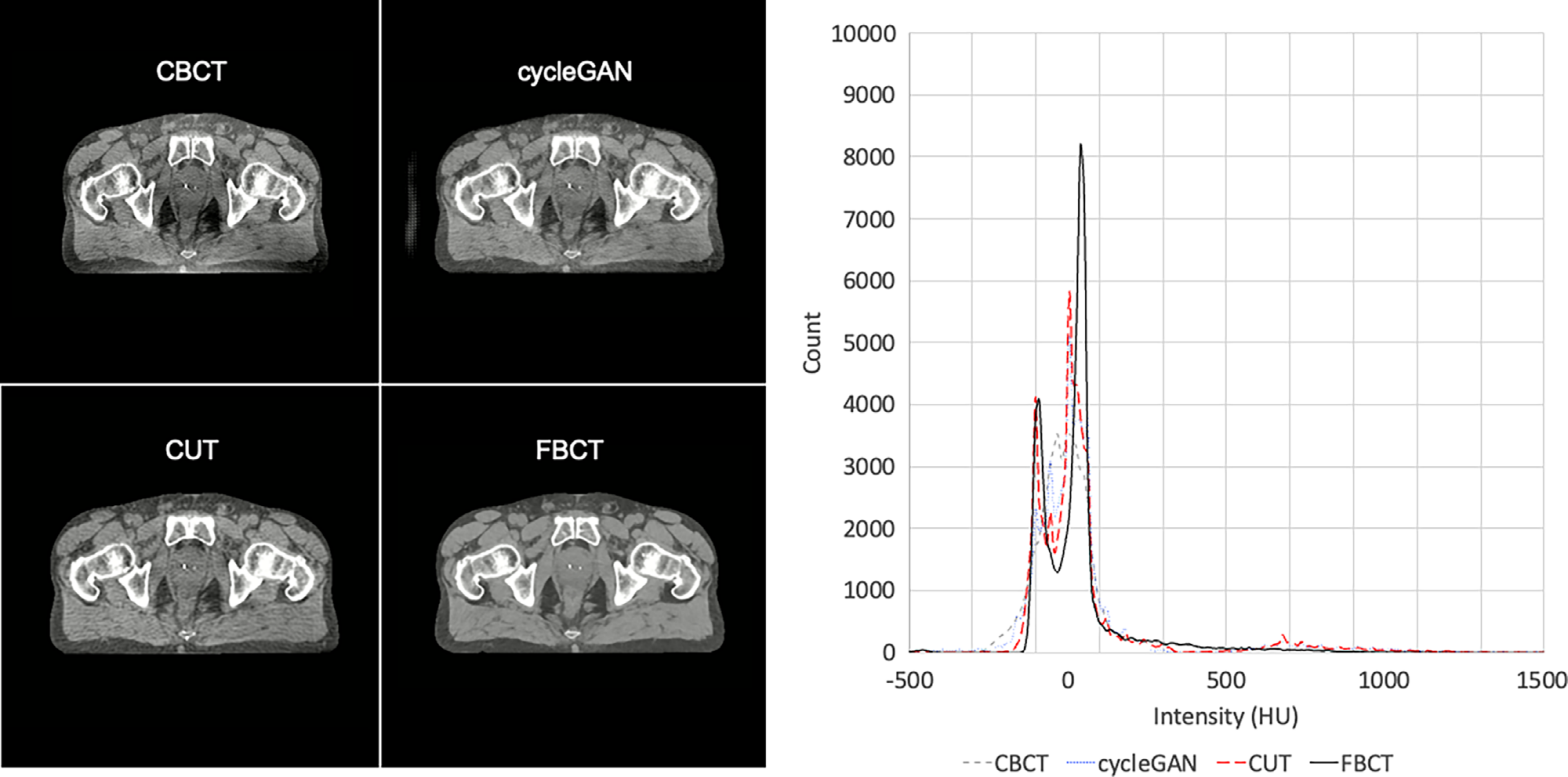
Comparison of results. Window: 500 Level: 20 for all images. Pixel histogram for displayed images presented at left top, linear scale. Pixel histogram for displayed images presented at left bottom, log scale.

Figure 2 demonstrates the visual results of the CUT model. Bone shadows and scatter are reduced while geometry and anatomy are preserved and HU fidelity is improved. Fiducial markers within the prostate are reconstructed with accuracy in position and size. Corresponding pixel value histograms are presented and demonstrate superior histogram matching of the CUT model relative to CycleGAN. The bimodal distribution of histogram values is lost with CycleGAN or CBCT, but preserved with CUT.

**FIGURE 3:**
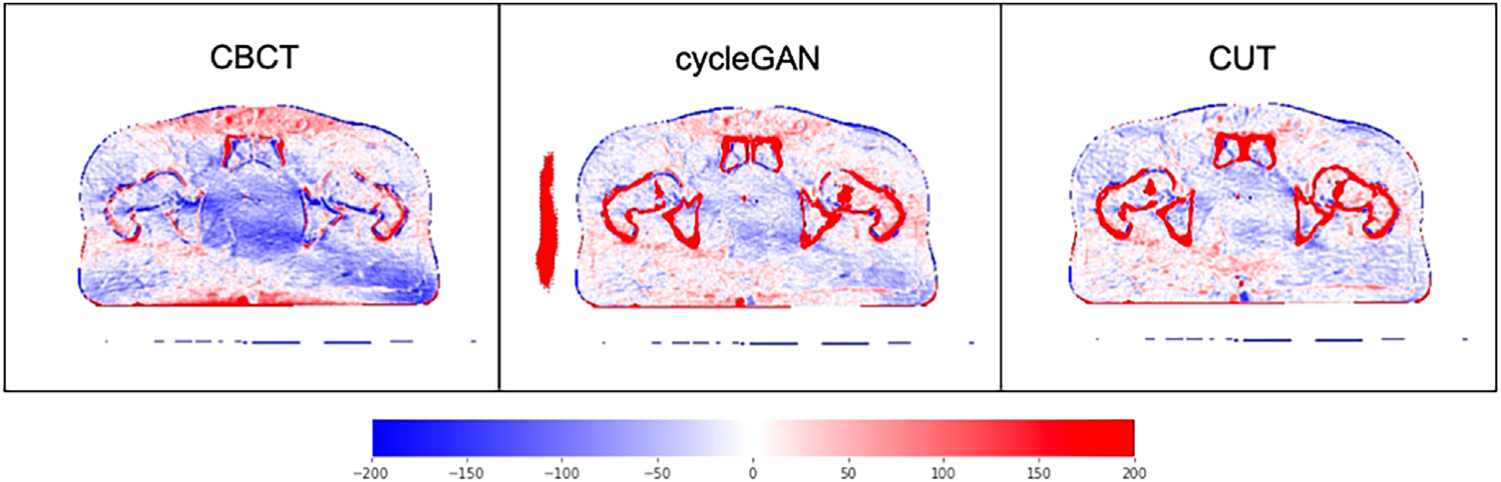
Subtraction plot relative to FBCT. Blue represents a negative difference, red is positive difference, white is zero difference.

Figure 3 is a subtraction plot with visualization of the differences between FBCT and CBCT, CycleGAN and CUT, respectively. Bone shadows cutting diagonally across the CBCT subtraction image are reduced by CycleGAN and CUT. Both CycleGAN and CUT demonstrate the greatest error at high contrast gradient edges such as the body surface or the interface between soft tissue and bone. CycleGAN creates an artifactual structure outside the body volume; CUT does not.

**FIGURE 4:**
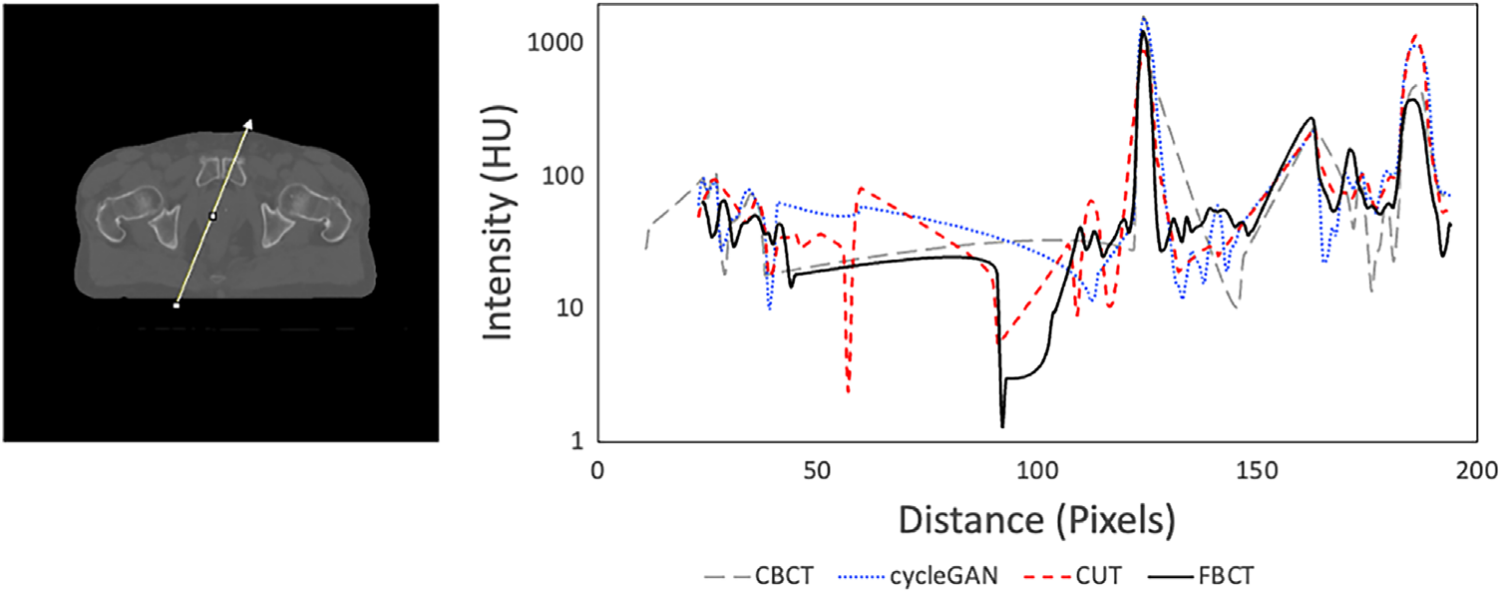
Profile plot along the ray depicted on the FBCT at left through muscle, fat, a fiducial, and bone. The peak at 160 pixels represents the fiducial marker, while those at 200 pixels and 220 pixels represent cortical bone.

The profile plot in Figure 4 further characterizes performance across muscle, fat, a fiducial marker, and bone. The peak at 125 pixels represents the fiducial marker while those at 160 pixels and 180 pixels represent cortical bone in the composite plot. CycleGAN more accurately reproduces the metal artifact associated with the fiducial as it appears in the FBCT; CUT reduces the severity of this artifact and improves the appearance of the surrounding soft tissue. CycleGAN and CUT both perform most poorly in bone, with the greatest deviation occurring in the area corresponding to cortical bone.

CUT is faster and lighter than CycleGAN. CycleGAN comprises four networks: two generators each with 11 378 000 parameters and two generators each with 2 800 00 parameters: a total of 28 286 000 parameters. CUT contains only one generator and discriminator as found in CycleGAN and deploys a MLP with 560 000 parameters for feature extraction from intermediate generator features, yielding a total parameter count of 14 703 000: approximately half that of CycleGAN (Table 1). As a result, CycleGAN computes on a single CT image slice over 0.33 s while CUT requires just 0.18 s.

**TABLE 1:** MAE, SSIM, RMSE, and FID are compared for the CBCT, CycleGAN, and CUT data relative to input FBCT.

Fold	1	2	3	4	5	
Samples	2184	2217	2218	2080	2080	
Mean Absolute Error (HU)						Mean (SE)
CBCT	35.24	18.13	26.61	16.42	15.7	22.42 (3.75)
cycleGAN	34.10	147.59	**23.70**	15.43	14.76	47.12 (25.36)
CUT	**32.80**	**15.24**	24.00	**14.28**	**11.30**	19.52 (3.94)
Structural Similarity Index						
CBCT	**0.77**	0.73	**0.80**	0.91	0.77	0.80 (0.03)
cycleGAN	0.76	0.00	0.79	0.91	0.77	0.65 (0.16)
CUT	0.75	**0.76**	0.79	**0.92**	0.77	0.80 (0.03)
Root Mean Square Error (HU)						
CBCT	**91.87**	**48.57**	**96.50**	**44.05**	**44.00**	65.00 (11.97)
cycleGAN	98.37	437.41	104.21	51.54	55.30	149.37 (72.81)
CUT	105.01	58.84	97.85	60.31	49.55	74.312 (11.28)
Fréchet Inception Distance						
CBCT	52.96	51.67	46.62	51.11	45.48	49.57 (1.48)
cycleGAN	**34.97**	241.2	32.32	37.34	33.77	75.92 (41.33)
CUT	55.26	**20.20**	**24.67**	**36.19**	**21.33**	31.53 (6.57)

Values in bold are best performance across methods. All values with statistical significance (*p* << 0.01).

**FIGURE 5:**
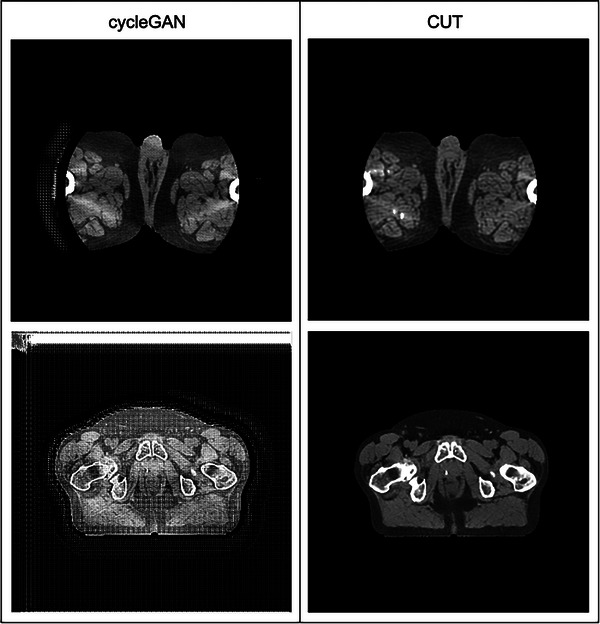
Modes of failure for cycleGAN. cycleGAN introduces artifactual structures when presented with a limited field of view (above) and performs poorly on Fold 2 of the dataset (below). Window: 500 Level: 20 for all images.

Failure modes are demonstrated for cycleGAN in Figure 5. The training dataset employed in this study underwent only minimal preprocessing. Truncated slices wherein the image is limited by CT field‐of‐view are present. The CUT model reproduced these without error while the CycleGAN model tended instead to generate false structures outside of the true field of view. CycleGAN also suffered mode collapse when training on data in the second fold. The CycleGAN model was re‐trained several times in an attempt to overcome this stochastic problem; however, the model ultimately failed to produce reasonable results on these data. The CUT model did not exhibit this behavior and trained without difficulty on all folds. Neither CycleGAN nor CUT were able to generate clinically useful images from the TCIA images after training on the institutional data.

MAE, RMSE, SSIM, and FID are compared for the CBCT, CycleGAN, and CUT data relative to input FBCT in Table 2. CUT demonstrates superior performance over CBCT and CycleGAN with respect to MAE as well as FID. MAE indicates pixel‐level correspondence to FBCT HU intensity values, making the synthetic outputs of CUT useful for dose calculations during ART. FID further demonstrates perceptual visual similarity with lesser values indicating greater similarity and is widely accepted as the gold standard for evaluating the quality of unsupervised image translation. CBCT, CycleGAN, and CUT perform most similarly on SSIM, indicating acceptable reproducibility of global structure.

**TABLE 2:** Summary of model size by component.

cycleGAN component	Parameters (millions)
Generator A	11.378
Generator B	11.378
Discriminator A	2.765
Discriminator B	2.765
Total	28.286

CUT component	Parameters (millions)
Generator	11.378
MLP	0.56
Discriminator	2.765
Total	14.703

CBCT is superior to CUT and CycleGAN with regard to RMSE. RMSE increases when single pixel values in the synthetic CT do not closely match the quality assurance FBCT. RMSE is most useful when large errors, however infrequent, are undesirable. Such isolated errors (single hot or cold pixels) will have little effect on visual quality and are unlikely to affect contour accuracy. For this reason, MAE is the more appropriate measure of error for the task of dose calculation in ART.

**FIGURE 6:**
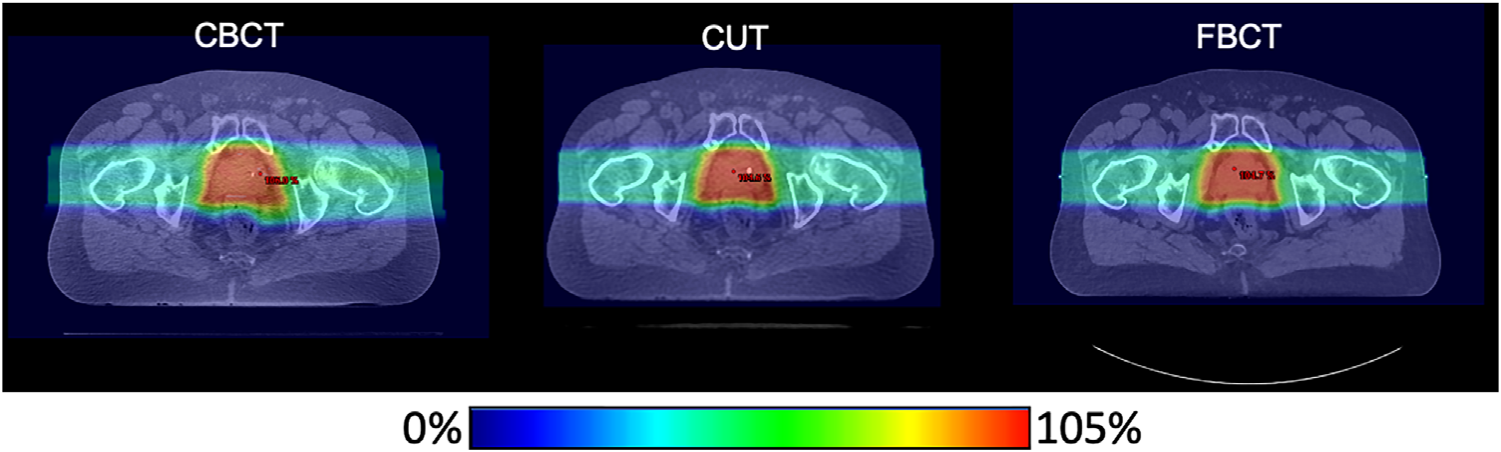
Comparison of dosimetric fidelity to ground truth same‐day quality assurance FBCT. Note the 50% isodose line reaches further left on the CBCT plan than the QACT or CUT plan.

Finally, CUT demonstrates greater fidelity to FBCT than CBCT at the time of adaptive treatment planning. Dose cloud artifacts created by CBCT error are absent in the plan based on the CUT output image and the dose distribution more nearly matches the ground truth same‐day FBCT (Figure 6).

## 4 DISCUSSION

We investigated a contrastive method to quantitatively improve CBCT images while simultaneously improving visual quality. The CUT method introduces a multi‐layer patch‐based approach that maximizes mutual information between image regions by drawing negatives from within the input image rather than from other images in the dataset. The model maintains anatomical boundaries while reducing artifacts and improving HU fidelity, spatial uniformity, artifact suppression and ultimately radiographic appearance. While CycleGAN also preserves HU fidelity, it fails in the preservation of anatomical boundaries, often introducing new artifactual structures into images. It does not perform as well in improving spatial uniformity, with a greater degree of scatter and bone shadow remaining in the output images. Furthermore, CycleGAN is incapable of coping with images with limited field of view, generating false structures outside of the image boundary.

We acquired additional test data from TCIA; however, when trained on the institutional dataset, neither CycleGAN nor CUT were able to generate clinically useful images from this new data acquired on a different scanner using an unfamiliar acquisition protocol. This is a result of the limited data available for model training rather than the model architecture. Relative to deformably‐registered planning CT images, same‐day quality assurance FBCT images represent a more accurate ground truth against which to evaluate model outputs. Unfortunately, data such as these are not easily collected. While this model cannot be shown to generalize to out‐of‐distribution data, it would be expected to do so given a large enough dataset of similar quality with a broader range of image acquisition parameters across several hardware equipment manufacturers.

A primary limitation of CycleGAN is instability during training. Mode collapse is a particular problem of GAN‐based methods wherein the model fails after falling into a local minimum distinct from the global minimum during optimization. This was encountered while training the CycleGAN model on the second fold. The CycleGAN model was re‐trained several times in an attempt to overcome mode collapse; however, the model ultimately failed to produce reasonable results on these data. The CUT model did not exhibit this behavior and trained without difficulty on all folds.

**FIGURE 7:**
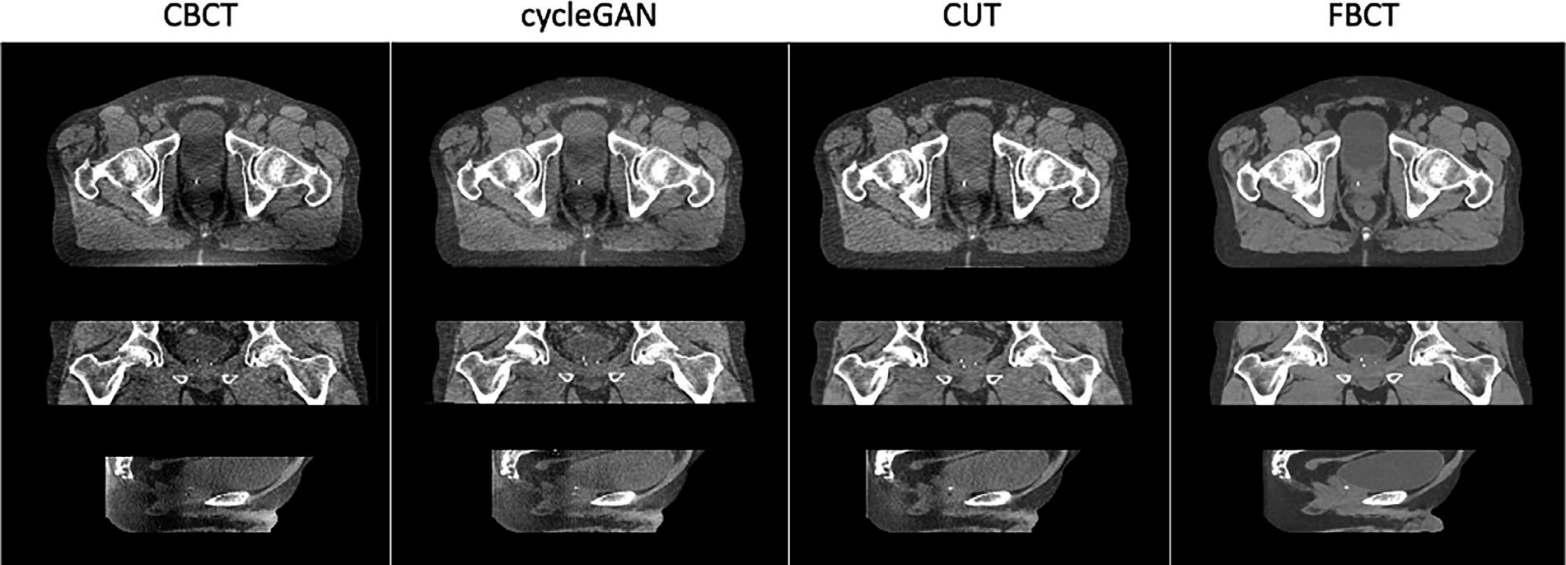
Comparison of image quality across reconstructed orthogonal planes for a single patient. Window: 500 Level: 20 for all images.

Training of 3D models is resource intensive. The models presented here were trained on axial 2D image slices. Translation accuracy is nevertheless preserved in orthogonal planes following volume reconstruction (Figure 7). Future study of three‐dimensional methods should balance gains in accuracy against losses in computational efficiency.

Large metal artifacts were not included in the training dataset; however, small fiducial markers were present in the prostate gland of some patients. While CycleGAN faithfully reproduced the associated metal artifacts, the CUT model instead attenuated them, improving the appearance of surrounding tissue. Whether CUT might be capable of reducing larger, more severe metal artifacts warrants further investigation.

We selected the male pelvis as the site of interest due to the variability in organ position within an otherwise well‐defined anatomic space with osseous boundaries. This represented a reasonable challenge for the models to overcome while making the acquisition of anatomically similar same‐day ground truth imaging feasible. We expect the CUT model to perform similarly well on other body sites, such as the head and neck or abdomen, given appropriate training data. We would further expect that the model described here would reasonably predict the output of additional scanner hardware, if presented with training data for that hardware.

## 5 CONCLUSION

The contrastive method investigated here is faster and more accurate than CycleGAN, requiring fewer networks and parameters to achieve superior performance. Computational speed and efficiency, as well as radiographic and dosimetric performance, are critical for the clinical deployment of this technology and particularly relevant to the specific application of online adaptive radiotherapy where the outputs must compute while the patient remains on the treatment table.

## AUTHOR CONTRIBUTIONS

Pretesh Patel, Sagar A. Patel, Karen Godette, and Ashesh B. Jani contributed patient data. Yang Lei, Shaoyan Pan, Tonghe Wang, and Xiaofeng Yang provided technical advice. Mosa Pasha and Kirk Luca assisted in the dosimetric experiments. In addition to these contributions, all authors critically revised the manuscript for intellectually important content and supplied final approval of the version to be published.

## CONFLICT OF INTEREST STATEMENT

The authors declare no conflicts of interest.

## Data Availability

The data that support the findings of this study are available from the corresponding author upon reasonable request.
